# Comparison of piezosurgery and conventional rotary instruments in schneider’s membrane sinus lifting: A pilot randomized trial

**DOI:** 10.4317/jced.57953

**Published:** 2021-08-01

**Authors:** Marcio Martins, Walbert-de Andrade Vieira, Luiz-Renato Paranhos, Rogério-Heládio-Lopes Motta, Carlos-Eduardo-Xavier-dos Santos-Ribeiro da Silva, Carvalho Rodriguez, Juliana-Cama Ramacciato

**Affiliations:** 1 Prevent Senior Institute, São Paulo, SP, Brazil; 2 Department of Restorative Dentistry, Endodontics division, Piracicaba Dental School, State University of Campinas – UNICAMP, Piracicaba, SP, Brazil; 3 Department of Community and Preventive Dentistry, School of Dentistry of Uberlândia, Federal University of Uberlândia, Uberlândia, MG, Brazil; 4 Department of Dentistry, São Leopoldo Mandic College, Campinas, SP, Brazil

## Abstract

**Background:**

The present study aimed to evaluate and compare the postoperative effects of the piezoelectric device and conventional rotary instruments in Schneider’s membrane sinus lifting procedure.

**Material and Methods:**

Twenty patients requiring bilateral maxillary bone graft augmentation in the posterior maxillary region were selected. Piezoelectric surgery was performed on one side and conventional surgery with a rotary diamond bur on the other. Postoperative pain, swelling, edema, and mouth opening were evaluated at one hour and two and seven days after the procedures. All variables were submitted to Friedman or Wilcoxon tests at a 5% significance level.

**Results:**

The comparison between groups showed that postoperative pain after one hour and two days was significantly lower (*p*< 0.05) in the piezoelectric device group. Regarding the edema, the results of both techniques were similar at all times assessed (*p*> 0.05). Piezosurgery was statistically associated (*p*< 0.05) with greater mouth opening only at the 48-hour evaluation.

**Conclusions:**

Osteotomy with a piezoelectric device causes less pain and greater mouth opening postoperatively compared with the conventional technique.

** Key words:**Piezosurgery, sinus lift, edema, pain, rotative instruments.

## Introduction

Dental implants gained space in dentistry for representing an effective and versatile method to rehabilitate simple to complex cases (1), returning quality to patients with tooth loss (2). However, an insufficient bone volume is a common clinical finding in rehabilitation procedures involving the posterior region of the maxilla and it is considered a complicating factor for installing implants in this region (3).

The most suiTable therapeutic proposal to reverse this situation is lifting the maxillary sinus by placing bone grafts on the floor of the sinus and below the sinus membrane (Schneiderian membrane) (4). This will increase the bone height of the maxillary ridge and allow placing dental implants in this region (5). One of the most used surgical techniques for this procedure is the lateral window technique, in which the space created between the residual maxillary ridge and elevated Schneiderian membrane is filled with a grafting material (5).

Conventionally, osteotomy using the lateral window technique is performed with burs (4). However, some postoperative complications are common after this technique, such as pain, ecchymosis, limited mouth opening, and edema (6). These complications are possibly due to high temperatures produced when cutting the jaw bone, which may induce marginal osteonecrosis and consequently impair post bone repair (7).

As an alternative, using the piezoelectric device in the lateral window technique was proposed to optimize the surgical procedure and minimize postoperative complications (8,9). *Pi*ezosurgery has the advantages of greater precision, effective selective cutting in the bone tissue, protection of the soft tissue, less bleeding in the surgical field, and faster bone tissue regeneration (10).

In this context, few studies in the literature compared the postoperative effects of maxillary sinus surgery using burs or piezoelectric devices (11-14). Thus, the present study aimed to compare two techniques of maxillary sinus osteotomy by lateral approach with either conventional burs or a piezoelectric device to quantify the differences in the criteria of edema, pain, mouth opening, and ecchymosis.

## Material and Methods

-Preliminary trial design

This study was submitted and approved by the Research Ethics Committee of the São Leopoldo Mandic Institute and Research Center (Campinas, Brazil) under protocol number 2.065.867 and followed the CONSORT guidelines for Pilots Trials (15). This is a prospective split-mouth randomized, double-blinded, pilot clinical trial conducted at a private clinic in São Paulo, Brazil, from 2016 to 2017.

The inclusion criteria for the study were men and women with partial or total edentulous region and indication for bilateral Schneider’s membrane elevation and grafting for posterior maxillary reconstruction; without systemic diseases contraindicating the procedure or oral and maxillofacial pathologies; not using any type of analgesic, antibiotic, or corticosteroid before the beginning of the study; and not presenting allergies to the drugs proposed for the clinical study.

The exclusion criteria were defined as: 1) patients with respiratory diseases and presence of chronic maxillary sinusitis; 2) consumption of 15 cigarettes per day and abuse of alcoholic beverages; 3) history of oral sinus communication before or at the time of surgery; and 4) inadequate psychological profile to follow the recommendations and cooperate with the data collection of the study.

After signing the Informed Consent Term, the patients were submitted to anamnesis; blood laboratory test (hemogram and coagulogram); evaluation of vital signs: blood pressure, partial oxygen saturation (SpO2), and heart rate, with the results considered baseline values for each patient; and panoramic radiographs and computed tomography of the maxilla for planning the cases.

-Surgical Procedures

The volunteers of the sample underwent two surgical procedures (right and left maxillary sinuses) and the order of the sides was defined by flipping a coin. The surgical technique (osteotomy with burs or piezoelectric device) was also chosen randomly by flipping a coin. In both moments, randomization was performed by a second researcher who was not involved in the study and did not know the clinical condition of the patient. Additionally, the patients were blinded to the technique used. All patients were subjected to a standardized surgical protocol by the same operator and the minimum interval of 30 days between the two interventions was respected.

The local anesthesia of both procedures was performed with 4% articaine hydrochloride with epinephrine 1: 100,000 (DFL, Rio de Janeiro, Brazil), respecting the volume of 3.6 ml, corresponding to two tubes, by the infiltrative technique in the deep groove region of premolars and maxillary molars. The incision was performed with a 15C modified Newman mucosal lamina with relaxing relief incisions in the canine and molar regions and detachment of the mucoperiosteal flap by a lateral approach to the wall of the maxillary sinus.

The osteotomy was performed with a rectangular aluminum surgical guide measuring 20x10 mm, on the lateral wall of the maxillary sinus, approximately 6 mm above the crest of the bone collar on both sides, according to the draw for selecting the conventional or piezoelectric technique, and with the recommended interval of 30 days between one side and the other.

The complete removal of the rectangular bone cover delimited by the osteotomy was initiated as well as the Scheneiderian membrane detachment procedures for the posterior insertion of biomaterial and adequacy of the collagen membrane. The surgery was finished by repositioning the flap and respective sutures in the region.

Regardless of the technique, the pharmacological protocol was the same for every surgery: 2 g of amoxicillin one hour before the procedure and continuity of 875 mg every 12 hours for five days; 8 mg of dexamethasone one hour before the procedure and 600 mg of ibuprofen every eight hours for two days. In addition to an intraoral antiseptic mouthwash for one minute with an aqueous solution of 0.12% chlorhexidine digluconate, there was an extraoral application of an aqueous solution of 2% chlorhexidine digluconate.

-Assessment of outcomes

All outcomes were assessed by one specialist in dental implantology who was blind to the technique used in the surgery.

The pain was assessed at one hour and two and seven days after the procedure using a visual analog scale (VAS) of 100 mm. On the VAS, the leftmost end represented the absence of pain (score 0) and the rightmost end indicated the most severe pain (score 10). The volunteers were instructed to return the forms in the session scheduled to remove the suture (seven days postoperative). The distance between the mark and the end with score 0 was measured by a digital caliper.

Edema was measured with a 3.0 silk thread and digital caliper before the procedure and at the second and seventh postoperative days, by determining pre-fixed points. This limited the area affected in the surgical procedure with a first plane defined by a line with boundaries between a point at the center of the tragus to a point in the center of the nasal border, a second plane by another line of intersection with boundaries between a point in the external corner of the eye to a point in the ipsilateral labial commissure, and a third plane from the center of the tragus to the labial commissure. Planes: 1) tragus to the nasal border; 2) external corner of the eye to the ipsilateral labial commissure, and 3) tragus to the labial commissure.

Mouth opening was measured according to a scale that shows the range of motion by the distance between the incisal edges of the right upper and lower incisors. A digital caliper was used for this measurement before surgery and in the second and seventh postoperative days.

-Data Analysis

The means and interquartile deviations were determined for each parameter of the study and control sides. All variables were submitted to the Friedman test to evaluate the effect of time in the same group and the Wilcoxon test to compare the two groups. All tests considered a significance level of 5% and were performed using the BioEstat 5.0 and GraphPad Prism 7.0 statistical packages.

## Results

**Figure 1:**
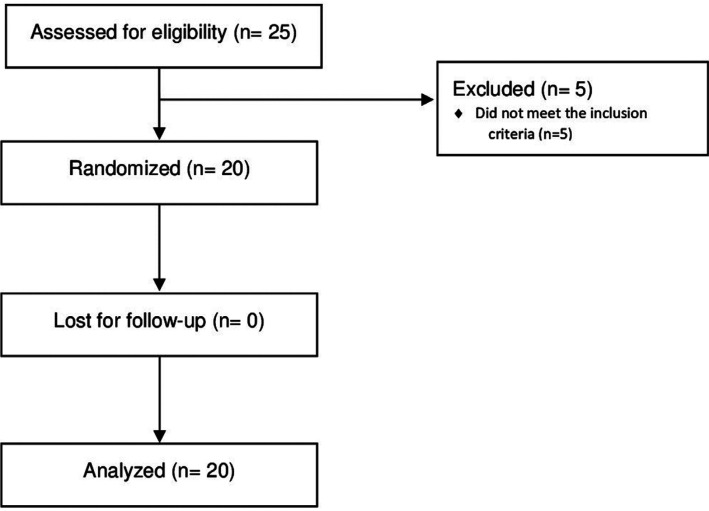
Flow diagram of a trial comparing rotary instruments and piezoelectric device for maxillary sinus lifting.

A total of 25 patients (seven men and 18 women; age range: 46 to 72 years) were recruited for the study. However, five patients were excluded for not meeting the inclusion criteria (Fig. 1).

**Figure 2:**
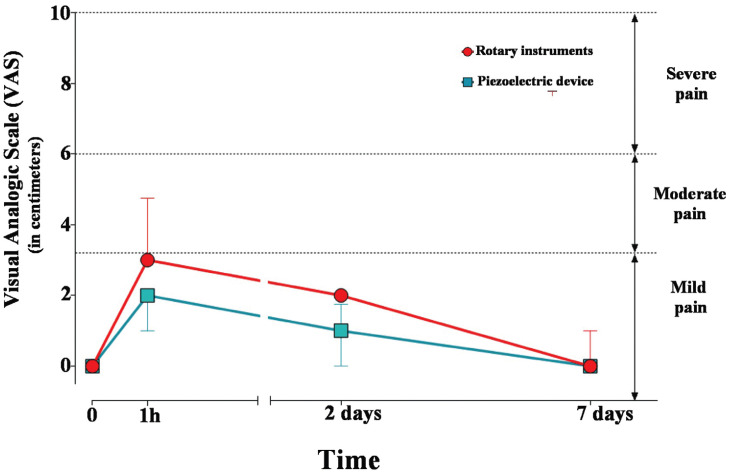
Median (interquartile deviation) of pain measured by VAS according to groups and assessment periods.

Both groups showed a significant increase (*p* <0.05) in pain after one hour and two days when compared to the preoperative evaluation. After seven days, the pain reported did not show statistically significant differences (*p*> 0.05) with the preoperative evaluation (Fig. 2). The comparison between groups showed that postoperative pain after one hour and two days was significantly greater (*p* <0.05) on the side that used burs for the osteotomy when compared to the side that used the piezoelectric device (Table 1).

**Table 1:**
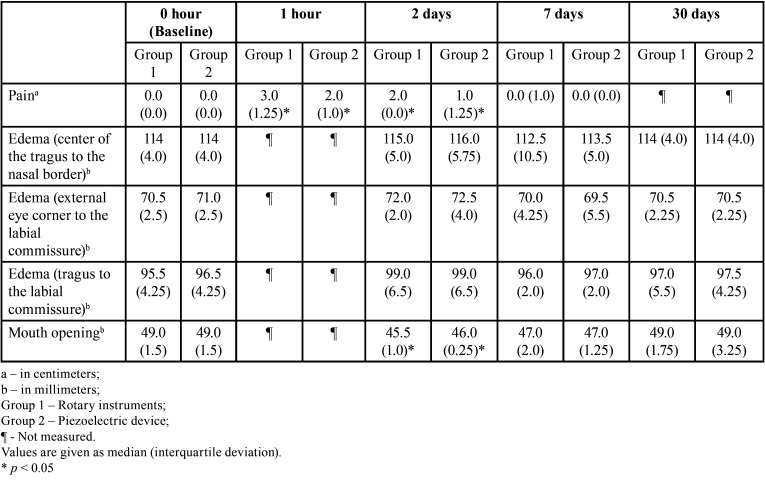
Results for pain, edema and mouth opening of each group in different time periods.

**Figure 3:**
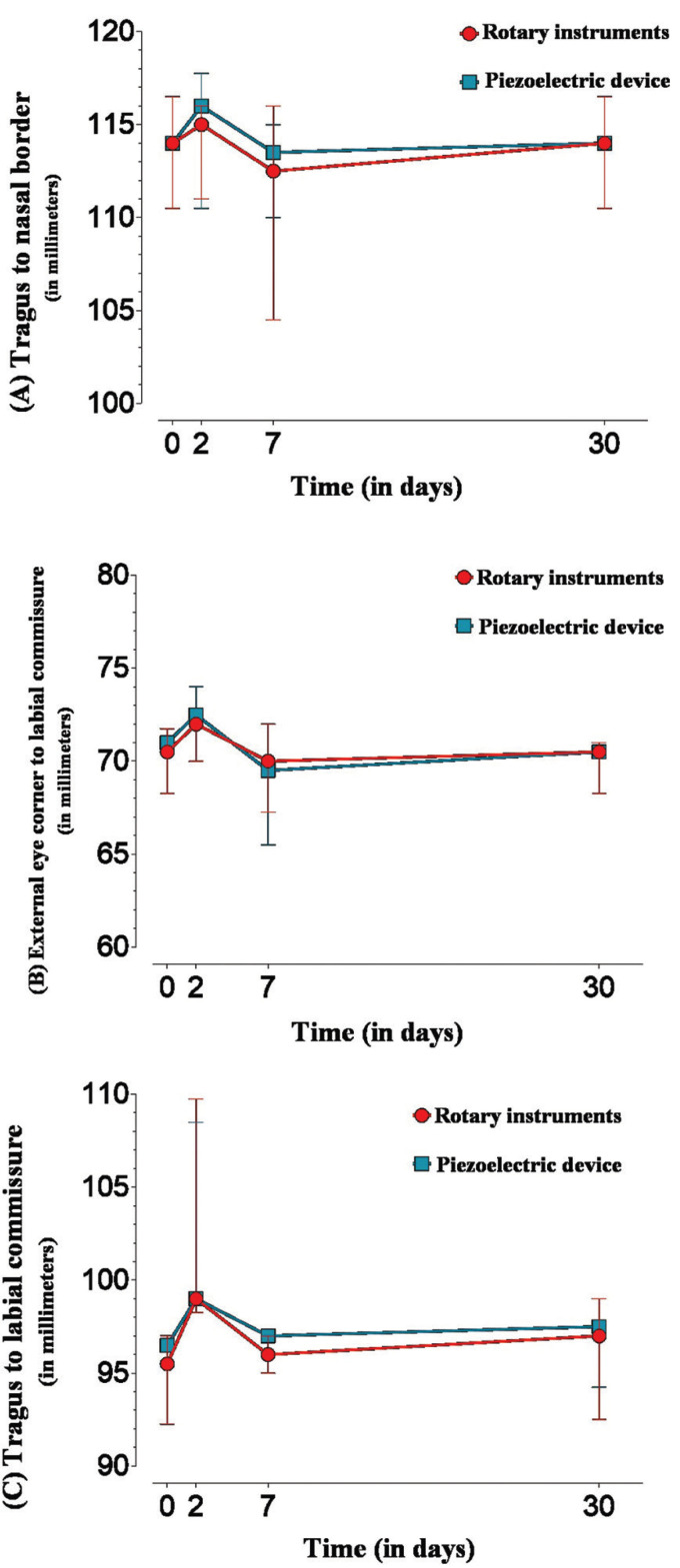
Median (interquartile deviation) of edema measured from (A) the center of the tragus to the nasal border; (B) from the external eye corner to the labial commissure; (C) the tragus to the labial.

The edema measured from the center of the tragus to the nasal border did not show a statistically significant difference between the burs and piezo groups in any period assessed (*p* > 0.05) (Fig. 3A). Thirty days after surgery, the patients showed no residual edema, with the values measured from the center of the tragus to the nasal border equal to the preoperative period (Fig. 3). When measured from the external corner of the eye to the labial commissure (Fig. 3B) or the center of the tragus to the labial commissure (Fig. 3C), the results of both techniques were similar at all times assessed (one hour, two days, seven days, and 30 days).

**Figure 4:**
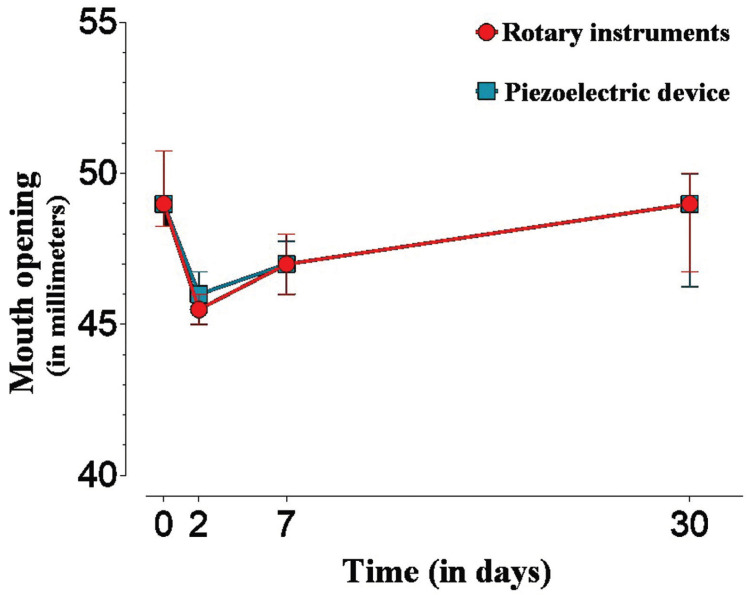
Median (interquartile deviation) of pain measured by VAS according to groups and assessment periods.

The data analysis revealed that, for both groups, there was a significant decrease (*p* <0.05) in mouth opening at two postoperative days, and this decrease was still maintained on the seventh day. At day 30, the values did not differ from the baseline values. Comparisons between the groups showed that, when operated with a piezoelectric device, patients presented statistically significant greater mouth opening only two days after surgery (*p* = 0.0180) (Fig. 4).

## Discussion

This pilot randomized trial aimed to assess the presence of pain, edema, and limited mouth opening in patients undergoing maxillary sinus lifting using rotary instruments or piezoelectric devices for osteotomy. The results showed that, when operated with piezoelectric devices, patients experience less pain and greater mouth opening within 48 hours after the procedure.

The piezoelectric device provides precise cutting in bone tissues, without damaging the noble structures (vessels, nerves, and mucous membranes), less heating during osteotomies, and a more favorable postoperative period (16). Histological and immunohistochemical studies have shown a reduced number of inflammatory cells, greater expression of bone morphogenetic proteins (BMP), and lower expression of pro-inflammatory cytokines after osteotomy with piezoelectric devices (17,18). This information helps to explain the lower pain intensity reported by patients when operated with piezoelectric devices compared to the conventional technique with rotary instruments. This finding agrees with that observed by other clinical study (13), which also found less pain intensity in the first 48 hours in patients treated with piezoelectric devices for maxillary sinus elevation.

Regarding edema, both techniques showed significant volume increases in the postoperative period compared to the preoperative period, within 48 hours, returning to normal levels after 30 days. Such findings agree with those found in other studies, considering that edema is caused by an inflammatory reaction in the tissues injured during surgical procedures (16). When compared to each other, the groups showed no significant differences for the size of the edema in any period investigated. These results are different from the study of Delilbasi *et al*. (13), which found statistically less edema in piezoelectric device group.

The decrease in mouth opening was also observed in the groups in the immediate postoperative period and after 48 hours, as described in other studies (16). When compared to each other, the groups showed no statistical difference in the immediate postoperative period, but after 48 hours the group operated with piezoelectric devices showed greater mouth opening than the group operated with rotary instruments. As with the results of pain intensity, the level of mouth opening seems to be associated with the intensity of the inflammatory process. Thus, the results obtained can be justified by the fact that piezoelectric devices cause less inflammation after surgery, especially after 48 hours, when the inflammatory process reaches its peak.

The main limitation of this study concerns the pilot character of the experimental design, which aimed to evaluate the applicability of the methodology proposed and the eligibility criteria, also serving as a parameter for calculating the sample of a future clinical trial. Another limitation concerns the small sample size, which implies less robust results. However, this is an original study and one of the few in the literature to compare the postoperative effects of piezosurgery for maxillary sinus lifting.

## Conclusions

Based on these preliminary results, it may be concluded that the piezoelectric osteotomy showed lower pain intensity in the initial evaluations (one hour and two days) and greater mouth opening on the second day. There was no difference in pain sensitivity and mouth opening between the techniques at seven and 30 days after surgery. As for edema, both techniques presented similar results in the postoperative period.
